# Describing dietary patterns of high-intensity functional training athletes: a cross-sectional study

**DOI:** 10.1080/15502783.2025.2550167

**Published:** 2025-08-26

**Authors:** Linh Nguyen, Kworweinski Lafontant, Claudia Gonzalez, Susan Kampiyil, Michelle Da Silva Barbera, Jack Livingston, Sofea Smith, Jeffrey R. Stout, David H. Fukuda

**Affiliations:** University of Central Florida, Physiology of Work and Exercise Response (POWER) Lab, Institute of Exercise Physiology and Rehabilitation Science, Orlando, FL, USA

**Keywords:** Nutrition, performance, dietary patterns, HIFT

## Abstract

**Background:**

High-Intensity Functional Training (HIFT; e.g. CrossFit^Ⓡ^, F45^Ⓡ^, Hyrox^Ⓡ^) is rapidly increasing in global participation. HIFT is a strenuous form of training that requires adequate dietary practices to optimize recovery and performance. However, newcomers to HIFT may not be aware of common dietary practices among current HIFT athletes as previous research on this topic is scarce. Therefore, the purpose of this study was to detail the dietary practices of HIFT athletes.

**Methods:**

We recruited 49 HIFT athletes (female = 24, male = 25; 37.8 ± 10.5 years; BMI = 25.9 ± 4.0 kg/m^2^) from HIFT gyms and social-media postings in the central Florida, USA, region for this cross-sectional study. We administered and supervised a paper version of the 16-item Rapid Eating Assessment for Participants-Shortened (REAP-S) questionnaire to assess self-reported dietary practices. Participant responses to REAP-S questions were coded as “usually/often,” “sometimes,” or “rarely/never.” The final question within the REAP-S questionnaire asked about the participants’ willingness to make changes to their dietary practices to improve their health, coded on a Likert-style 1–5 range (1 = “very willing,” 5 = “not at all willing”). Descriptive statistics are presented; no inferential tests were performed.

**Results:**

All participants report cooking at home. Most rarely dine out (*n* = 38, 78%) or skip breakfast (*n* = 32, 65%). Nearly all usually/often or sometimes consume ≥8 oz (~227 g) of lean meats per day (*n* = 46, 94%) and rarely consume processed meats (*n* = 32, 65%). Approximately 55% (*n* = 27) report adding discretionary fats (e.g. butter) to meals, and 57% (*n* = 28) rarely snack on sweets or fried foods. Participants usually/often or sometimes consume at least two daily servings of fruits (*n* = 29, 59%), whole-grains/dairy (*n* = 28, 57%), and vegetables (*n* = 24, 49%). Most participants abstain from >16 oz (~473 mL) of sugar-sweetened beverages per day (*n* = 46, 94%). Regarding their willingness to adopt healthier dietary practices, participants averaged 1.4 ± 0.6 out of 5, indicating a high readiness to change to improve their health.

**Conclusions:**

HIFT athletes typically favor homemade meals, frequent lean-meat intake, and limit sugar-sweetened beverage consumption. Most HIFT athletes consume at least two daily servings of fruits, whole-grains, and dairy while avoiding ultra-processed foods. However, only ~50% meet the U.S. recommendation of ≥2 servings of vegetables per day, suggesting room for improvement – particularly given their strong willingness to change. While this study was limited by self-report and further multi-site research is warranted, participation in HIFT appears to be concomitant with evidence-based dietary practices for performance.

